# Circulating Chemerin and Its Kinetics May Be a Useful Diagnostic and Prognostic Biomarker in Critically Ill Patients with Sepsis: A Prospective Study

**DOI:** 10.3390/biom12020301

**Published:** 2022-02-12

**Authors:** Irene Karampela, Gerasimos Socrates Christodoulatos, Natalia Vallianou, Dimitrios Tsilingiris, Evangelia Chrysanthopoulou, George Skyllas, Georgios Antonakos, Ioanna Marinou, Evaggelos Vogiatzakis, Apostolos Armaganidis, Maria Dalamaga

**Affiliations:** 1 Second Department of Critical Care, Attikon General University Hospital, Medical School, National and Kapodistrian University of Athens, 1 Rimini St., Haidari, 12462 Athens, Greece; eyacriss@yahoo.gr (E.C.); georg.skyllas@gmail.com (G.S.); aarmag@med.uoa.gr (A.A.); 2 Department of Biological Chemistry, Medical School, National and Kapodistrian University of Athens, Mikras Asias 75, Goudi, 11527 Athens, Greece; gerchristod82@hotmail.com (G.S.C.); madalamaga@med.uoa.gr (M.D.); 3 First Department of Internal Medicine, Evangelismos General Hospital, 45-47 Ipsilantou Str., 10676 Athens, Greece; natalia.vallianou@hotmail.com; 4 First Department of Propaedeutic Internal Medicine, Medical School, National and Kapodistrian University of Athens, Laiko General Hospital, 17 St Thomas Street, 11527 Athens, Greece; tsilingirisd@gmail.com; 5 Laboratory of Clinical Biochemistry, Medical School, National and Kapodistrian University of Athens, Attikon General University Hospital, 1 Rimini Street, Haidari, 12462 Athens, Greece; georgiosantonakos@yahoo.gr; 6 Laboratory of Microbiology, Sotiria Athens General Hospital, 152 Mesogeion Avenue, 11527 Athens, Greece; ioannachond@yahoo.gr (I.M.); vogia2@gmail.com (E.V.)

**Keywords:** adipokine, adipose tissue, biomarker, chemerin, critically ill, mortality, sepsis, septic shock

## Abstract

Chemerin, a novel adipokine, is a potent chemoattractant molecule with antimicrobial properties, implicated in immune responses. Our aim was to investigate circulating chemerin and its kinetics, early in sepsis in critically ill patients and its association with severity and prognosis. Serum chemerin was determined in a cohort of 102 critically ill patients with sepsis during the first 48 h from sepsis onset and one week later, and in 102 age- and gender-matched healthy controls. Patients were followed for 28 days and their outcomes were recorded. Circulating chemerin was significantly higher in septic patients at onset compared to controls (342.3 ± 108.1 vs. 200.8 ± 40.1 μg/L, *p* < 0.001). Chemerin decreased significantly from sepsis onset to one week later (342.3 ± 108.1 vs. 308.2 ± 108.5 μg/L, *p* < 0.001), but remained higher than in controls. Chemerin was higher in patients presenting with septic shock than those with sepsis (sepsis onset: 403.2 ± 89.9 vs. 299.7 ± 99.5 μg/L, *p* < 0.001; one week after: 374.9 ± 95.3 vs. 261.6 ± 91.9 μg/L, *p* < 0.001), and in nonsurvivors than survivors (sepsis onset: 427.2 ± 96.7 vs. 306.9 ± 92.1 μg/L, *p* < 0.001; one week after: 414.1 ± 94.5 vs. 264.2 ± 79.9 μg/L, *p* < 0.001). Moreover, patients with septic shock and nonsurvivors, presented a significantly lower absolute and relative decrease in chemerin one week after sepsis onset compared to baseline (*p* < 0.001). Based on ROC curve analyses, the diagnostic performance of chemerin (AUC 0.78, 95% CI 0.69–0.87) was similar to C-reactive protein (CRP) (AUC 0.78, 95% CI 0.68–0.87) in discriminating sepsis severity. However, increased chemerin at sepsis onset and one week later was an independent predictor of 28-day mortality (sepsis onset: HR 3.58, 95% CI 1.48–8.65, *p* = 0.005; one week after: HR 10.01, 95% CI 4.32–23.20, *p* < 0.001). Finally, serum chemerin exhibited significant correlations with the severity scores, white blood cells, lactate, CRP and procalcitonin, as well as with biomarkers of glucose homeostasis, but not with cytokines and soluble urokinase-type plasminogen activator receptor (suPAR). Circulating chemerin is increased early in sepsis and its kinetics may have diagnostic and prognostic value in critically ill patients. Further studies are needed to shed light on the role of chemerin in sepsis.

## 1. Introduction

Chemerin, a recently characterized adipokine, is a biologically active molecule exerting cytokine-like actions, secreted mainly by the adipose tissue [1]. Chemerin is also produced by the liver, the skin, the pancreas, the adrenal gland, the kidney and the lung, as it is expressed by numerous cells besides adipocytes: hepatocytes, fibroblasts, and epithelial cells [2]. Prechemerin (the inactive form of chemerin) is activated by the inflammatory and coagulation serine proteases through cleavage of its C-terminus [3,4].

As an adipokine, chemerin is involved in adipogenesis and glucose homeostasis by regulating metabolism in the adipose tissue, the liver and the skeletal muscles, and it is associated with obesity and insulin resistance [1,5,6,7]. It is also involved in myogenesis and angiogenesis, with implications for tumor growth [8,9,10,11,12]. However, chemerin had been initially described as an immunomodulatory molecule, which acts as a potent chemotactic factor for macrophages, natural killer cells and immature dendritic cells [4,9,13,14]. These immune cells express the chemerin receptor CMKLR1 (chemokine-like receptor 1) on their surface and respond to chemerin binding with integrin activation, calcium signaling and alteration of their functional status, which result in chemotaxis to sites of tissue injury or inflammation and in the initiation of an appropriate immune response (pro-inflammatory or anti-inflammatory), depending on the local milieu [9,13,14]. Experimental evidence supports a versatile role for chemerin in coordinating early immune responses, similar to a context dependent role of chemerin in various cancers [15]. Chemerin has been shown to stimulate macrophage adhesion to extracellular matrix proteins and adhesion molecules in vitro, in a murine experimental study [16]. This finding suggests that chemerin may enhance inflammation through recruitment and retention of macrophages at the sites of inflammation. Chemerin has also been shown to inhibit neutrophil recruitment and secretion of pro-inflammatory cytokines and chemokines in an animal model of acute lung injury, demonstrating potent anti-inflammatory actions [17]. Additionally, in this study chemerin increased recruitment of alveolar macrophages, which are implicated in the regulation of local immune homeostasis by exerting immunosuppressive actions. Thus, chemerin may play a key role in the regulation of immune responses, presenting variable pro- or anti-inflammatory activity in different clinical scenarios. Furthermore, experimental evidence suggests that inflammatory mediators such as interleukin 1β (IL-1β), tumor necrosis factor alpha (TNFα), interferon γ (IFNγ) and lipopolysaccharide (LPS) induce chemerin expression by adipocytes, hepatocytes and epithelial cells [9,18,19]. Finally, chemerin has been shown to exert antimicrobial properties, inhibiting bacterial growth after activation by host-derived, as well as pathogen-derived proteases [20,21,22,23,24].

Animal studies have demonstrated a multifaceted role of chemerin in the pathogenesis of various inflammatory diseases: a protective role in animal models of LPS-induced lung injury, viral pneumonia, and peritonitis, but a detrimental effect in chronic obstructive pulmonary disease and autoimmune encephalomyelitis [2,9,17,25,26,27]. Clinical studies have also revealed that chemerin is increased in inflammatory and autoimmune diseases [6,28,29]. Circulating chemerin was recently shown to increase in an animal model of peritoneal infection and a high-fat diet, as well as in patients with peritoneal sepsis, being associated with biomarkers of glucose homeostasis, insulin resistance, sepsis severity and prognosis [30].

Various adipokines, i.e., hormones derived mainly from the adipose tissue, exert immunomodulatory actions, being implicated in the inflammatory response during sepsis [31,32,33,34]. Previous studies have highlighted that circulating adipokines are altered during sepsis in critically ill patients and may serve as diagnostic and prognostic biomarkers [35,36,37,38,39,40,41,42,43]. However, chemerin has not been thoroughly studied in sepsis. We hypothesized that circulating chemerin is altered during sepsis and may be associated with its severity and prognosis. Therefore, the aim of our study was to: (1) evaluate serum chemerin levels in critically ill patients with sepsis at onset compared to healthy controls; (2) investigate chemerin’s kinetics during the first week of sepsis; (3) examine its relationship with clinical and inflammatory biomarkers; and (4) explore the diagnostic and prognostic value of chemerin and its kinetics.

## 2. Materials and Methods

### 2.1. Study Population and Protocol

This was a prospective observational study that was conducted in the mixed adult intensive care unit (ICU) of a tertiary teaching hospital from August 2013 to July 2015. The study protocol conformed to the Declaration of Helsinki and its successive amendments and was approved by the Scientific and Ethics Committee of the hospital (#587/10-04-2013). All study participants or their next of kin provided informed consent.

**Figure 1:**
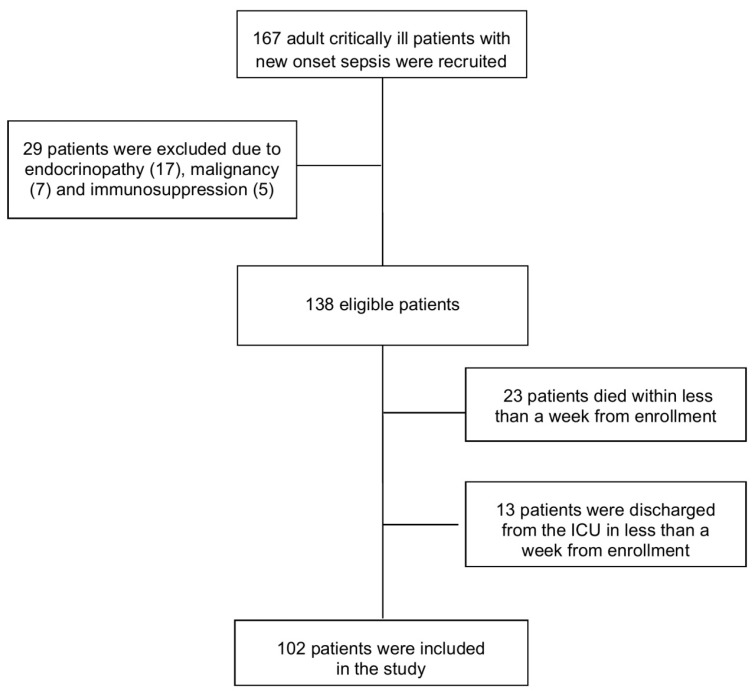
Flowchart of the study population.

Consecutive adult (>18 years) critically ill patients with new onset sepsis (within 48 h) were enrolled in the study. Patients were enrolled according to the previously established consensus definition for sepsis [44]. However, after completion of enrollment, a new consensus for sepsis and septic shock definitions was reached, named SEPSIS-3 [45]. In order to correctly present the characteristics of our study population according to currently used criteria, we recategorized the study population based on prospectively recorded clinical and laboratory data, i.e., the SOFA score, lactate, hypotension, need for fluid resuscitation and vasopressors and signs of organ dysfunction. Since our study population included only critically ill patients, all patients presented with organ dysfunction at sepsis onset. Therefore, no patient was excluded from the study after applying SEPSIS-3 definition criteria. Patients who were discharged from the ICU or died in less than a week from inclusion in the study were excluded. Patients younger than 18 years of age, pregnant patients, those receiving total parenteral nutrition or had a history of endocrinopathy, liver disease, malignancy or immunosuppression were also excluded from the study. A total of one hundred and sixty seven consecutive critically ill patients with new onset sepsis were admitted to the ICU during the study period. After excluding 65 patients, according to the pre-specified exclusion criteria, we included 102 patients (57 men, 45 women, mean age: 64.7 ± 15.6 years) (Figure 1). Demographic, clinical and routine laboratory data were recorded. All patients were followed for 28 days after enrollment.

The control group consisted of 102 healthy subjects (57 men, 45 women, mean age: 66.4 ± 10.3 years). These were healthy, non-pregnant adults with no history of endocrinopathy, liver disease, malignancy or immunosuppression, who visited the outpatient laboratory department for routine checkup. Healthy controls were selected to be gender- and age-matched (± 5 years) to every eligible case enrolled. The detailed study protocol has been previously published elsewhere [36,38,43].

### 2.2. Laboratory Analysis

Upon enrollment, blood samples were collected from patients and healthy controls, as well as from patients one week after enrollment. Serum was retrieved from whole blood specimens by centrifugation and was stored at −80 °C. Determination of chemerin concentration in serum was conducted in duplicate by an immunoenzymatic method with a commercially available enzyme-linked immunosorbent assay (ELISA) kit (Human Chemerin, BioVendor, Brno, Czech Republic, RD191136200R), with a sensitivity of 0.1 μg/L, <8.3% intra-assay coefficient of variation (CV), and <7% inter-assay CV. It is important to mention that this immunoassay cannot distinguish between the various forms of inactive or active chemerin. Homeostasis model assessment score of insulin resistance (HOMA-IR) was calculated by the formula: fasting serum insulin (μU/mL) × fasting serum glucose (mmol/L)/22.5. Determination of metabolic, inflammatory and coagulation parameters, interleukins (IL) IL-1β, IL-6, IL-10 and soluble urokinase-type plasminogen activator receptor (suPAR) was conducted by ELISA (eBiosciences, San Diego, CA, USA and suPARnostic™, ViroGates, Lyngby, Denmark), as previously described [36,38,46,47,48,49,50,51].

### 2.3. Statistical Analysis

We analyzed data employing the chi-square test for categorical variables; *t*-test and paired *t*-test for normally distributed variables; and Mann–Whitney U and Wilcoxon matched pair tests for non-normally distributed variables. The normality hypothesis was tested using the Shapiro–Wilk test. Spearman correlation coefficients (r) were used as measures of correlation for continuous variables. Survival curves were derived using the Kaplan–Meier method, and comparisons were performed employing the log rank test. The discriminating power of used biomarkers in distinguishing sepsis category and mortality prediction was evaluated by receiver operating characteristic (ROC) curves. Finally, in order to discern the independent laboratory predictors of 28-day mortality, a multivariate Cox-regression analysis was performed, adjusting for acute physiology and chronic health evaluation (APACHE) II as designed a priori, and statistically significant inflammatory biomarkers. Due to the small number of nonsurvivors, it is impossible to include all significant clinical and laboratory variables in the regression model. APACHE II represents the most widely used ICU mortality prediction score, taking into account 12 admission physiologic variables, including among others, patient’s age, white blood count, creatinine, electrolytes, etc., [52]. Based on previous studies on adipokines [11,36,43], we calculated that we required a total sample size of at least 200 participants to achieve 95% power at the 0.05 level of significance, in order to detect a 25 μg/L difference in circulating chemerin. A two-sided *p* value of less than 0.05 was considered significant. The statistical package IBM-SPSS^®^ version 24 for Windows (IBM Corp., Armonk, NY, USA) was used for the analysis of the study.

## 3. Results

### 3.1. Baseline Demographic, Clinical and Laboratory Data in Patients and Controls

The baseline characteristics and main laboratory data of patients and controls are summarized in Table 1. Upon enrollment, sixty patients had sepsis and 42 had septic shock, according to the SEPSIS-3 criteria. The patients consisted of 61 medical cases (60%), 29 surgical cases (28%) and 12 trauma cases (12%). The site of infection causing sepsis was pulmonary (35%), abdominal (24%) and other (pyelonephritis, complicated skin and soft tissue infections, bacteremia, meningitis, and infectious endocarditis). The causative pathogens were Gram negative bacteria in 60%, Gram positive bacteria in 23% and fungi in 17% of the 60 cases where a pathogen was isolated. Thirty patients (29.4%) died within 28 days from inclusion in the study. Patients and controls did not differ in age, gender and BMI, as shown in Table 1. However, hemoglobin concentration and platelets were lower, while white blood cells were higher in patients than controls. Regarding metabolic biomarkers, serum albumin and total protein were lower, and serum glucose, insulin and HOMA-IR were higher in patients than controls. The coagulation indices were also significantly higher in patients compared to controls.

### 3.2. Circulating Chemerin in Patients and Controls

**Figure 2:**
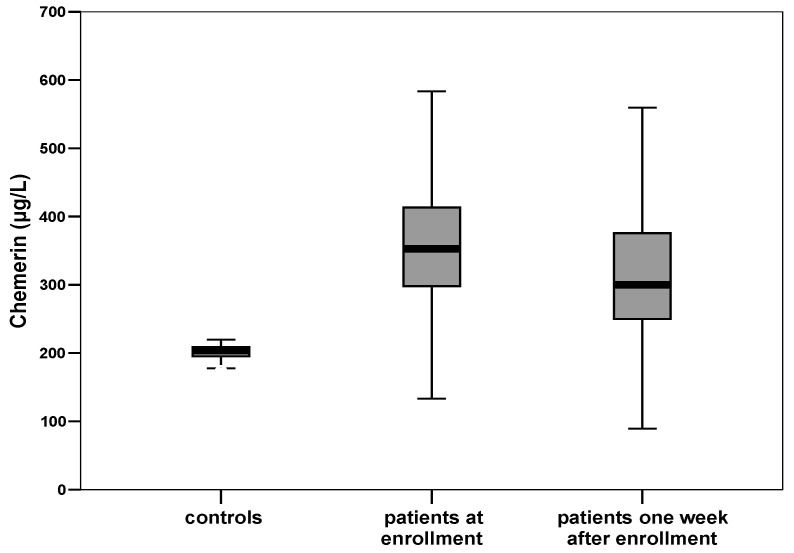
Box plots of circulating chemerin in healthy controls and in septic patients at enrollment and one week after. At enrollment, septic patients present a significantly higher mean level of chemerin than controls (342.3 ± 108.1 μg/L vs. 200.8 ± 40.1 μg/L, *p* < 0.001). Circulating levels of chemerin in septic patients decrease significantly one week after enrollment (342.3 ± 108.1 μg/L vs. 308.2 ± 108.5 μg/L, *p*< 0.001).

**Figure 3:**
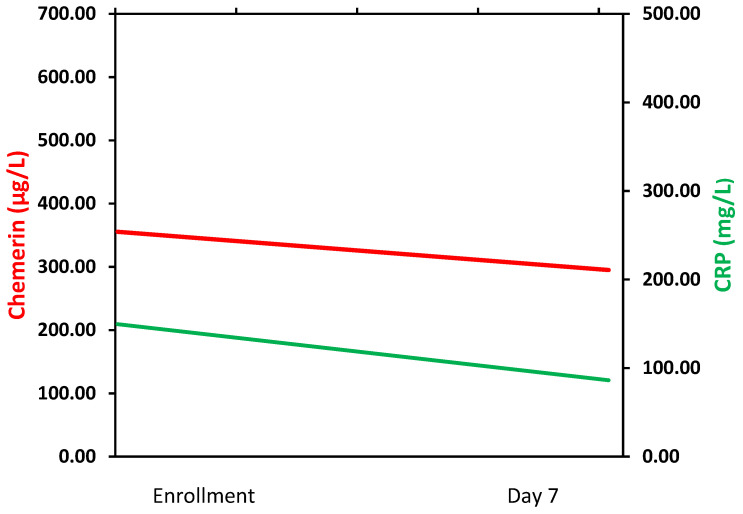
Graphical trend of changes over time of circulating chemerin and C-reactive protein (CRP) (from enrollment to day 7).

At enrollment, critically ill patients with sepsis had a significantly higher serum chemerin than healthy controls (342.3 ± 108.1 vs. 200.8 ± 40.1 μg/L, *p* < 0.001) (Table 1). During the first week from sepsis onset, serum chemerin presented a significant decrease in all patients (342.3 ± 108.1 vs. 308.2 ± 108.5 μg/L, *p* < 0.001) (Figure 2). However, serum chemerin was still significantly higher in septic patients than controls, one week after sepsis onset (*p* < 0.001). Chemerin exhibited similar kinetics with C-reactive protein (CRP). Figure 3 portrays the graphical trend of changes over time of circulating chemerin and CRP (from enrollment to day 7).

### 3.3. Circulating Chemerin According to Sepsis Severity

**Figure 4:**
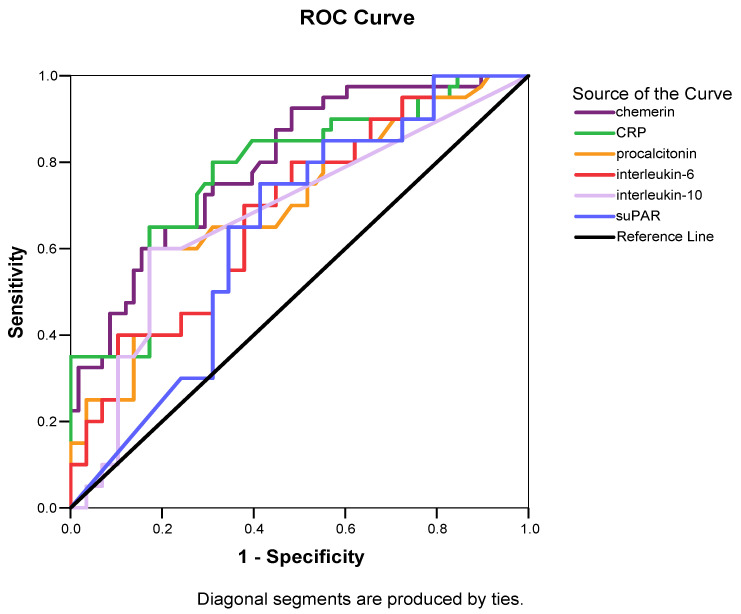
Area under the receiver operating characteristic curve (AUROC) distinguishing sepsis from severe sepsis in 102 patients with sepsis. Based on Table 3, circulating chemerin (AUROC > 0.78) and C-reactive protein (AUROC > 0.78) at enrollment outperform procalcitonin (AUROC > 0.71), IL-6 (AUROC > 0.69), IL-10 (AUROC > 0.68) and suPAR (AUROC > 0.64) in distinguishing sepsis from septic shock.

Serum chemerin was higher in patients presenting with septic shock at enrollment compared to those with sepsis, both at sepsis onset (*p* < 0.001) and one week later (*p* < 0.001) (Table 2). Chemerin kinetics were similar in both groups of patients, presenting a significant decrease during the first week from sepsis onset. However, patients with sepsis presented a significantly higher decrease in serum chemerin compared to patients with septic shock (Δchemerin% 12.9 ± 5.8% vs. 7.4 ± 6.4%, *p* < 0.001). ROC curves were generated for chemerin and other inflammatory biomarkers which differed significantly between patients with sepsis and those with septic shock at enrollment (Figure 4). Circulating chemerin and CRP at sepsis onset outperformed procalcitonin, IL-6, IL-10 and suPAR in discriminating sepsis from septic shock (Table 3).

### 3.4. Circulating Chemerin and Mortality

**Figure 5:**
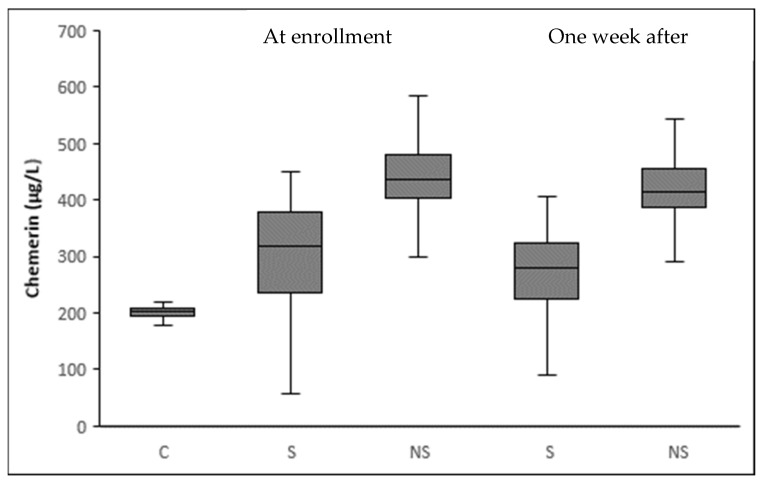
Box plots of circulating chemerin at enrollment and one week after stratifying by 28-day mortality status. At enrollment, septic patients present a significantly higher mean level of chemerin (342.3 ± 108.1 μg/L vs. 200.8 ± 40.1 μg/L, *p* < 0.001) than controls (C). Circulating levels of chemerin are significantly higher in nonsurvivors (NS) than survivors (S) septic patients both at enrollment (427.2 ± 96.7 μg/L vs. 306.9 ± 92.1 μg/L, *p* < 0.001) and one week after (414.1 ± 94.5 μg/L vs. 264.2 ± 79.9 μg/L, *p* < 0.001). Additionally, the decrease in serum chemerin one week after enrollment is significant in both groups of patients (*p* < 0.001 for both S and NS). Finally, the mean difference between serum chemerin at enrollment and one week after is higher in S than NS (42.7 ± 22.2 μg/L vs. 13.2 ± 11.3 μg/L, *p* < 0.001).

**Figure 6:**
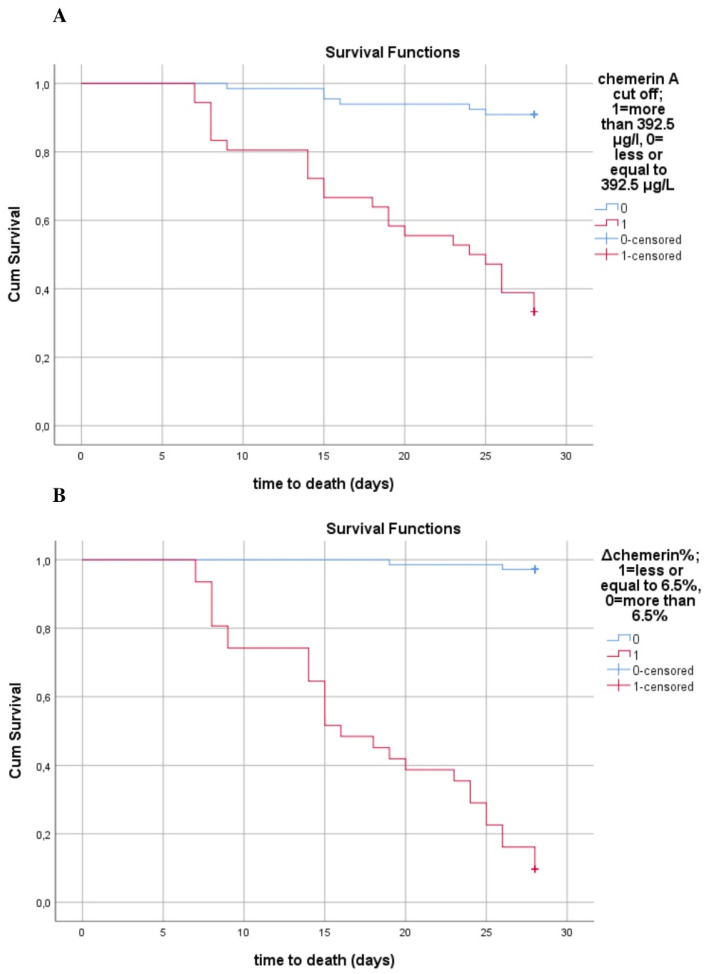
Kaplan–Meier estimates of mortality in 102 septic patients (**A**) Kaplan–Meier estimates of mortality based on serum chemerin at enrollment cutoff values obtained via ROC analysis (log rank test: 42.15, *p* < 0.001). (**B**) Kaplan–Meier estimates of mortality based on absolute percentage change of serum chemerin from baseline cutoff values obtained via ROC analysis (log rank test: 111.4, *p* < 0.001).

Circulating chemerin was significantly higher in nonsurvivors compared to survivors at enrollment as well as one week later, as shown in Figure 5 (*p* < 0.001). Regarding kinetics, both groups of patients presented a significant decrease in chemerin levels during the first week of sepsis (*p* < 0.001). However, survivors exhibited a greater mean decrease in chemerin (42.7 ± 22.2 μg/L vs. 13.2 ± 11.3 μg/L, *p* < 0.001), and a greater percentage change from baseline (Δchemerin% 13.8 ± 5.1% vs. 3.1 ± 2.4%, *p* < 0.001) compared to nonsurvivors The Kaplan–Meier survival curves showed that patients with lower circulating chemerin at sepsis onset presented improved survival, with the cutoff value of chemerin being 392.5 μg/L (Figure 6A). Additionally, patients with a higher percentage change (decrease) compared to baseline in chemerin levels during the first week of sepsis had improved survival at 28 days, with the cutoff value of percentage change estimated at 6.5% (Figure 6B).

Unadjusted Cox regression analyses showed that chemerin at enrollment (HR: 1.013, 95% C.I. 1.009–1.018, *p* < 0.001) and one week after was significantly associated with mortality at 28 days after sepsis onset (HR: 1.015, 95% C.I. 1.01–1.02, *p* < 0.001). After adjustment for the APACHE II score, higher chemerin at enrollment was an independent predictor of 28-day mortality (HR 3.58, 95% C.I. 1.48–8.65, *p* = 0.005). One week after enrollment, higher chemerin was also an independent predictor of mortality (HR: 10.01, 95% C.I. 4.32–23.20, *p* < 0.001) (Table 4). Noteworthy, IL-6 at enrollment and one week after, but not CRP at enrollment, was also independently associated with mortality.

### 3.5. Correlations between Chemerin and Other Biomarkers

**Figure 7:**
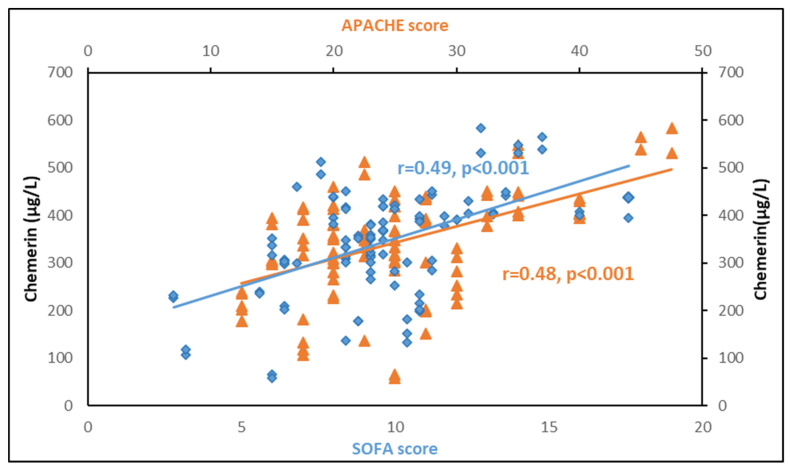
Circulating chemerin is significantly associated with APACHE II and SOFA scores at sepsis onset in 102 critically ill septic patients.

Table 5 depicts Spearman correlations between serum chemerin and biomarkers of sepsis. Circulating chemerin presented significant positive correlations with the severity scores APACHE II and SOFA both at sepsis onset and one week after, being stronger at sepsis onset (Figure 7). Chemerin also exhibited positive correlations with white blood cells and lactate, but a negative correlation with total protein and albumin at enrollment and one week later. Additionally, chemerin correlated significantly with glucose, insulin and HOMA-IR at enrollment. Of note, chemerin presented a strong positive correlation with BMI in the control group (r = 0.51, *p* < 0.001). However, this correlation is of borderline significance in septic patients (Table 5). Chemerin was significantly associated with prothrombin and activated partial thromboplastin only at enrollment, but not with fibrinogen. Regarding inflammatory biomarkers, chemerin showed a significant positive correlation with CRP and procalcitonin only at sepsis onset. This correlation was stronger with CRP than procalcitonin. Finally, we did not find any significant association of chemerin with IL-1β, IL-6, IL-10 or suPAR.

## 4. Discussion

In this prospective study, we explored circulating chemerin, a novel adipokine, and its kinetics in critically ill patients with sepsis, during the first week from sepsis onset. We found that chemerin was significantly higher compared to healthy controls, with more elevated values in septic shock than sepsis and in nonsurvivors than survivors. Regarding kinetics, serum chemerin decreased one week after sepsis onset in all patients, but this decrease was greater in those presenting with sepsis than septic shock, and in those who survived for 28 days than nonsurvivors. The key finding of our study is that higher circulating chemerin at sepsis onset, as well as sustained elevation of chemerin during the first week of sepsis was associated with the severity of sepsis and the 28-day mortality. Of note, circulating chemerin at sepsis onset and one week later was an independent predictor of 28-day mortality after adjustment for APACHE II score, which is a well-established mortality prediction score for critical illness [52].

To the best of our knowledge, this is the first study to investigate circulating chemerin and its kinetics during the early phase of sepsis in critically ill patients. There is only one small prospective study of 14 patients with peritoneal sepsis that demonstrated increased circulating chemerin compared to controls, and association of chemerin with sepsis severity, in agreement with our findings [30]. In the same study, the findings were also confirmed in a murine model of sepsis. Moreover, the study included a second independent cohort of 37 patients with peritoneal sepsis and showed that increased serum chemerin is associated with a worse outcome in septic patients without stress hyperglycemia. Finally, this study reported an association of serum chemerin with disturbed glucose homeostasis and insulin resistance in septic patients [30]. In line with the previous findings, we found a significant correlation of chemerin with the severity scores APACHE II and SOFA and also with glucose, insulin and HOMA-IR at sepsis onset. However, in our study we further showed that chemerin at sepsis onset and one week after, as well as its kinetics were associated with the severity and 28-day mortality of sepsis. Furthermore, we demonstrated that chemerin may predict 28-day mortality independently of the APACHE II score.

**Figure 8:**
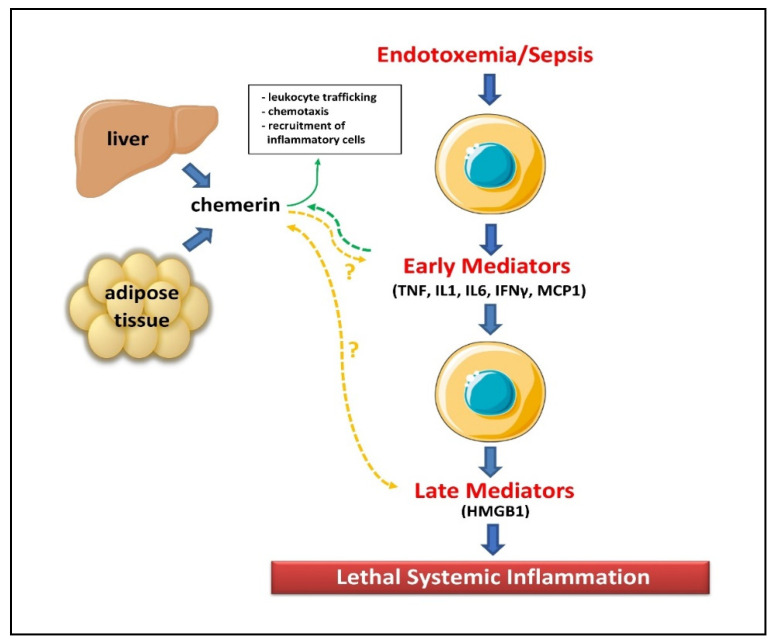
Actions of chemerin and cytokines that are secreted during sepsis. Abbreviations: HMGB1, high mobility group box 1; IFN, interferon; IL, interleukin; MCP1, monocyte chemoattractant protein-1; TNF, tumor necrosis factor. (Images of the liver and macrophages are originated from the free medical website http://smart.servier.com/ (accessed on 14 December 2021) by Servier licensed under a Creative Commons Attribution 3.0 Unported License).

Our findings are in line with experimental evidence regarding the immunomodulatory actions of chemerin. Inflammatory mediators such as the pro-inflammatory cytokines IL-1β, TNFα, and IFNγ, secreted during sepsis, have been shown to induce chemerin expression in adipocytes as well as other cells [9,18,19,53]. Moreover, in vitro studies have highlighted that lipopolysaccharide (LPS), the most potent microbial factor implicated in the pathogenesis of sepsis, may modulate chemerin activity [53]. In particular, LPS upregulates the expression of CC chemokine receptor-like 2 (CCRL2), which is a high-affinity chemerin receptor in endothelial cells, leading to increased chemerin levels [54]. Additionally, sepsis-induced activation of platelets and the coagulation cascade may also enhance chemerin secretion and activation by multiple mechanisms. Platelets have been shown to store chemerin and release it when stimulated, while circulating plasma proteases such as carboxypeptidases, along with coagulation associated proteases such as thrombin, plasmin, tissue-type and urokinase-type plasminogen activator, activate chemerin by proteolytic cleavage [13,55]. Moreover, activated leukocytes, particularly polymorphonuclear leukocytes, rapidly respond at the onset of inflammation, releasing a wide range of proteases such as elastase, tryptase, chymase, matrix metalloprotease and cathepsins, which all promote activation of chemerin [1,13]. Subsequently, bioactive chemerin further induces the chemotaxis and infiltration of antigen-presenting cells (macrophages, dendritic cells and natural killer cells) to the sites of inflammation, modulating the development and evolution of local and systemic inflammatory response in sepsis [14] (Figure 8).

Our study showed that higher chemerin at sepsis onset as well as one week after is associated with sepsis severity and outcome. This finding suggests that higher circulating chemerin during the first week from sepsis onset indicates the persistence of inflammation leading to aberrant immune response driving the mortality. Instead, lower chemerin at sepsis onset, as well as a greater decrease one week later, denotes the resolution of sepsis and a favorable outcome. We also found a significant positive correlation of chemerin with glucose, insulin and HOMA-IR at sepsis onset. This finding confirms the known association of chemerin with insulin resistance and impaired glucose tolerance [5,6,7]. Additionally, evidence suggests that insulin may represent a positive feedback for chemerin. Indeed, besides inflammatory factors, insulin has been shown to significantly upregulate chemerin production in human adipose tissue and serum [56].

Finally, we demonstrated a significant correlation of chemerin with CRP and procalcitonin, but not IL-1β, IL-6, IL-10 or suPAR. Circulating chemerin is subject to proteolytic processing of the C-terminal by a plethora of proteases, which produce various isoforms of chemerin, each exhibiting a differential affinity to the respective receptors and diverse activity [4,9,20]. Therefore, the post-secretory processing of inert chemerin may regulate its bioactivity through a complex interaction of all chemerin variants. In our study, we determined total chemerin, and could not differentiate inert chemerin from its active isoforms. This may explain the observed discrepancy in the association of chemerin with other inflammatory biomarkers.

Our study has shown that chemerin performed similarly to CRP and better than other biomarkers for the early discrimination of sepsis severity, while chemerin at enrollment, but not CRP, predicted 28-day mortality independently of the APACHE II score. These findings suggest that chemerin may prove to be of diagnostic and prognostic value in sepsis in combination with other biomarkers. Until today, no biomarker has been proven adequate to be used alone for sepsis. Larger prospective studies are needed to corroborate our findings, to further investigate the role of chemerin, and to explore its diagnostic and prognostic value in sepsis as well as its incorporation in an algorithm combining important laboratory and clinical parameters.

The main strengths of our study include: (1) the prospective design; (2) the age- and gender-matching of cases and controls; (3) the sufficient statistical power to show significant results between cases and controls; and (4) the multivariate analysis taking into account important confounding factors. Nevertheless, the study presents certain limitations. We used healthy outpatients, and not critically ill patients without sepsis as the control group. Since sepsis is very common in critical illness, enrollment of non-septic critically ill patients as controls would have posed practical difficulties. Additionally, critical illness is characterized by multiorgan dysfunction due to organ injury (such as acute lung and kidney injury) that may affect many inflammatory biomarkers, which might obscure the results of the study. We excluded patients who were either discharged from the ICU or succumbed before completing one week from the enrollment to the study. This resulted in the exclusion of cases at the extremes of severity of sepsis, constituting a selection bias. However, excluding both the less and the more severe cases might have counterbalanced any such effect. Furthermore, the included patients comprise a highly representative sample of septic patients, as this is reflected in the reported mortality. Among patients presenting with sepsis (*n* = 60), 6 died within 28 days (mortality rate 10%), and among those presenting with septic shock (*n* = 42) 24 died (mortality rate 57%). The outcome in our study sample is in agreement with the reported mortality for sepsis and septic shock of large patient cohorts used in the development of the recent SEPSIS-3 definitions [45]. Also, the results of this single-centre study may not be generalized to other critically ill populations with sepsis. Finally, despite the careful design and the adjusted statistical analyses, residual confounding cannot be excluded.

## 5. Conclusions

In a prospective study in critically ill patients with sepsis, we found that circulating chemerin was significantly increased at sepsis onset and one week after, compared to age- and gender-matched healthy controls. We also showed that chemerin was associated with severity and 28-day mortality of sepsis. Finally, we found that higher chemerin and its lower kinetics during the first week of sepsis were independent predictors of 28-day mortality. Our findings suggest that circulating chemerin and its kinetics may be a useful diagnostic and prognostic biomarker in sepsis. Further prospective studies are warranted to elucidate the pathophysiologic mechanisms of chemerin in sepsis.

## Figures and Tables

**Table 1:** Clinical and laboratory baseline variables* of patients and controls (*n* = 204).

Parameters	Patients (n = 102)	Controls (n = 102)	*p*-Value
Age ^a^, years	64.7 ± 15.6	66.4± 10.3	0.35
Gender, male, n (%)	57 (55.9)	57 (55.9)	0.56
BMI ^a^, kg/m^2^	29.9 ± 8.5	28.1 ± 5.01	0.06
Septic shock, n (%)	42 (41.2)	-	
Death before 28 days, n (%)	30 (29.4)	-	
*Severity scoring*			
APACHE II ^a^	23 ± 7.2	-	
SOFA ^a^	10 ± 3.3	-	
*Hematologic parameters*			
Hemoglobin ^a^, g/L	93 ± 20	147.9 ± 16.3	<0.001
White Blood Cells ^a^ × 10^9^/L	14.1 ± 8.4	6.97 ± 1.8	<0.001
Platelets ^a^ × 10^9^/L	216.2 ± 118.8	243.8 ± 46.9	0.03
*Metabolic biomarkers*			
Albumin ^a^, g/L	24.6 ± 5.9	46.7 ± 5.6	<0.001
Lactate ^b^, mmol/L	2.1 (1–9)	-	
Creatinine ^a^, μmol/L	123.76 ± 70.72	74.26 ± 12.38	0.08
Total Protein ^a^, g/L	50 ± 9	71± 4.2	<0.001
Glucose ^a^, mmol/L	7.97 ± 2.9	5.32 ± 1.16	<0.001
Insulin ^b^, pmol/L	197.9 (88.2–402.8)	73.13 (22.2–430.2)	<0.001
HOMA-IR ^b^	8.9 (3.24–34.5)	2.3 (0.65–23.5)	<0.001
*Coagulation indices*			
Prothrombin time ^a^, sec	14.3 ± 4.7	11.9 ± 0.8	<0.001
aPTT ^a^, sec	38.9 ± 9.4	34.4 ± 7.3	<0.001
Fibrinogen ^a^, μmol/L	14.49 ± 5.26	9.06 ± 1.3	<0.001
*Inflammatory biomarkers*			
CRP ^b^, mg/L	132 (7–431)	-	-
Procalcitonin ^b^, μg/L	0.9 (0.1–100)	-	-
IL-1β ^b^, ng/L	5.9 (5.9–206)	-	-
IL-6 ^b^ ng/L	27.4 (6–444)	-	-
IL-10 ^b^, ng/L	5 (5–300)	-	-
suPAR ^b^, μg/L	13 (2.1–16.8)	-	-
Chemerin ^a^, μg/L	342.3 ± 108.1	200.8 ± 40.1	<0.001

* Values of normally distributed variables are reported as mean ± SD, and those of non-normally distributed variables are reported as median (range). Abbreviations: APACHE II, acute physiology and chronic health evaluation score; aPTT, activated partial prothrombin time; BMI, body mass index; CRP, C-reactive protein; HOMA-IR, homeostasis model assessment of insulin resistance; IL, interleukin; SOFA, sequential organ failure assessment score; suPAR, soluble urokinase-type plasminogen activator receptor. ^a^ Mean ± SD, ^b^ Median, range.

**Table 2:** Major laboratory biomarkers* of patients with sepsis and septic shock, at baseline and one week after enrollment (*n* = 102).

	At Enrollment			One Week after Enrollment		
Laboratory Biomarkers	Sepsis (*n* = 60)	Septic Shock (*n* = 42)	*p*-Value	Sepsis (*n* = 60)	Septic Shock (*n* = 42)	*p*-Value
*Hematologic indices*						
White Blood Cells ^a^ × 10^9^/L	12.5 ± 5.9	16.3 ± 10.7	0.02	8.5 ± 3.2	16.2 ± 11.1	<0.001
Platelets ^a^ × 10^9^/L	230.4 ± 117.6	195.8 ± 118.8	0.15	252.7 ± 120.3	174.6 ± 97.9	0.001
*Markers of organ dysfunction*						
Albumin ^a^, g/L	26 ± 5.6	22.6 ± 5.7	0.004	25.1 ± 4.8	22.5 ± 4.2	0.005
Lactate ^b^, mmol/L	1.2 (1–5)	2.4 (2.1–9)	<0.001	1 (1–2.7)	1.9 (0.7–19)	<0.001
*Inflammatory biomarkers*						
CRP ^b^, mg/L	89 (7–218)	174 (36–431)	<0.001	55 (8–282)	101 (13–253)	0.01
Procalcitonin ^b^, μg/L	0.7 (0.09–47.7)	4.8 (0.14–100)	0.002	0.5 (0.06–15)	1.4 (0.14–83)	0.001
IL-1β ^b^, ng/L	5.9 (5.9–207)	8.8 (5.9–44.8)	0.18	17 (5.9–499)	8.8 (5.9–45)	0.13
IL-6 ^b^, ng/L	16.5 (6–385)	74.4 (10–444)	0.001	25 (4.6–419)	20.5 (6–487)	0.34
IL-10 ^b^, ng/L	5 (5–300)	6.9 (5–87)	0.001	5 (5–300)	5 (5–66)	0.02
suPAR ^b^, μg/L	10.5 (2.2–16.8)	14.1 (4.4–16.8)	0.04	11.3 (2.6–16.8)	12.9 (5.2–16.8)	0.68
Chemerin ^a^, μg/L	299.7 ± 99.5	403.2 ± 89.9	<0.001	261.6 ± 91.9	374.9 ± 95.3	<0.001

*Values of normally distributed variables are reported as mean ± SD, and those of non-normally distributed variables are reported as median (range). Abbreviations: CRP, C-reactive protein; IL, interleukin; suPAR, soluble urokinase-type plasminogen activator receptor. ^a^ Mean ± SD, ^b^ Median, range.

**Table 3:** Receiver operator characteristic curve analysis to ascertain the optimum cutoff value of major circulating biomarkers at enrollment in order to discern sepsis from septic shock in 102 patients with sepsis.

Biomarkers	AUC (95% CI)	*p*-Value	Sensitivity	Specificity	Youden’s Index	Cutoff Value	Positive Predictive Value	Negative Predictive Value
Chemerin	0.78 (0.69–0.87)	<0.001	71.4%	70%	0.41	362.8 μg/L	62.5%	77.8%
CRP	0.78 (0.68–0.87)	<0.001	80%	69%	0.49	132 mg/L	64.4%	83.1%
Procalcitonin	0.71 (0.60–0.81)	0.001	60%	82.8%	0.43	4.30 μg/L	70.9%	74.7%
IL-6	0.69 (0.58–0.79)	0.001	70%	62.1%	0.32	24.50 ng/L	56.4%	74.7%
IL-10	0.68 (0.57–0.79)	0.003	60%	82.8%	0.43	5.88 ng/L	70.9%	74.7%
suPAR	0.64 (0.53–0.75)	0.02	75%	58.6%	0.34	11.79 μg/L	55.9%	77%

Abbreviations: AUC, area under the curve; CI, confidence interval; CRP, C-reactive protein; IL, interleukin; suPAR, soluble urokinase-type plasminogen activator receptor.

**Table 4:** Multivariate Cox regression analyses results for the independent laboratory predictors of mortality (expressed as quartiles) adjusting for APACHE II score in 102 septic patients.

	b	SE_b_	Wald	df	*p*-Value	HR	95% for C.I.
*Independent Inflammatory Laboratory Predictors at Enrollment*							
Chemerin	1.27	0.45	8.01	1	0.005	3.58	1.48–8.65
CRP	0.23	0.19	1.42	1	0.23	1.26	0.86–1.85
IL-6	0.38	0.18	4.32	1	0.03	1.46	1.02–2.09
APACHE II	1.37	0.49	7.81	1	0.005	3.94	1.51–10.29
*Independent Inflammatory Laboratory Predictors at Day 7*							
Chemerin	2.30	0.43	28.8	1	<0.001	10.01	4.32–23.20
CRP	0.41	0.21	3.96	1	0.04	1.51	1.006–2.27
IL-6	0.83	0.21	16.02	1	<0.001	2.29	1.53–3.45
APACHE II	1.01	0.27	14.63	1	<0.001	2.78	1.64–4.66

Abbreviations: APACHE II, acute physiology and chronic health evaluation II; b, regression coefficient; CI, confidence interval; CRP, C-reactive protein; df, degree of freedom; HR, hazard ratio; IL-6, interleukin 6; SE_b_, standard error of b.

**Table 5:** Spearman correlation coefficients of circulating chemerin with severity scores and laboratory biomarkers in septic patients at enrollment and one week after (*n* = 102).

Variables	At Enrollment		One Week after Enrollment	
	r	*p*	r	*p*
*Clinical scoring*				
APACHE II	0.48	<0.001	0.36	<0.001
SOFA	0.49	<0.001	0.35	<0.001
*Hematologic indices*				
Hemoglobin	0.01	0.93	−0.09	0.38
White Blood Cells	0.24	0.01	0.21	0.03
Platelets	−0.06	0.55	−0.38	<0.001
*Biomarkers of organ dysfunction*				
Albumin	−0.28	<0.01	−0.31	0.001
Lactate	0.57	<0.001	0.35	<0.001
Creatinine	0.31	0.001	0.16	0.11
*Metabolic parameters*				
Total protein	−0.32	0.001	−0.46	<0.001
Glucose	0.26	0.02	-	-
Insulin	0.43	0.03	-	-
HOMA-IR	0.47	<0.001	-	-
BMI	0.19	0.06	-	-
*Coagulation biomarkers*				
Prothrombin time	0.34	<0.001	0.11	0.27
aPTT	0.39	<0.001	0.15	0.13
Fibrinogen	0.04	0.67	−0.11	0.28
*Inflammatory biomarkers*				
CRP	0.44	<0.001	−0.06	0.57
Procalcitonin	0.226	0.02	0.16	0.11
IL-1β	0.08	0.4	−0.11	0.3
IL-6	0.14	0.16	−0.10	0.32
IL-10	0.16	0.12	−0.08	0.45
suPAR	0.09	0.39	0.08	0.45

Abbreviations: APACHE II, acute physiology and chronic health evaluation score; aPTT, activated partial thromboplastin time; BMI, body mass index; CRP, C-reactive protein; HOMA-IR, homeostasis model assessment of insulin resistance; IL, interleukin; SOFA, sequential organ failure assessment score; suPAR, soluble urokinase-type plasminogen activator receptor.

## Data Availability

Data to support the findings of this study are available upon reasonable request.

## Abbreviations

APACHE, acute physiology and chronic health evaluation; aPTT, activated partial thromboplastin time; BMI, body mass index; CCRL2, C-C chemokine receptor-like 2; CI, confidence interval; CMKLR1, chemokine-like receptor 1; CRP, C-reactive protein; CV, coefficient of variation; ELISA, enzyme linked immunosorbent assay; HOMA-IR, homeostasis model assessment of insulin resistance; HR, hazard ratio; ICU, intensive care unit; IFNγ, interferon γ; IL, interleukin; LPS, lipopolysaccharide; ROC, receiver operating characteristic; SOFA, sequential organ failure assessment score; suPAR, soluble urokinase-type plasminogen activator receptor; TNFα, tumor necrosis factor alpha.

## References

[1] Helfer G., Wu Q.-F. (2018). Chemerin: A multifaceted adipokine involved in metabolic disorders. J. Endocrinol..

[2] Bondue B., Wittamer V., Parmentier M. (2011). Chemerin and its receptors in leukocyte trafficking, inflammation and metabolism. Cytokine Growth Factor Rev..

[3] Du X.-Y., Leung L.L. (2009). Proteolytic regulatory mechanism of chemerin bioactivity. Acta Biochim. et Biophys. Sin..

[4] Mattern A., Zellmann T., Beck-Sickinger A.G. (2014). Processing, signaling, and physiological function of chemerin. IUBMB Life.

[5] Goralski K.B., McCarthy T.C., Hanniman E.A., Zabel B.A., Butcher E.C., Parlee S.D., Muruganandan S., Sinal C.J. (2007). Chemerin, a Novel Adipokine That Regulates Adipogenesis and Adipocyte Metabolism. J. Biol. Chem..

[6] Ernst M.C., Sinal C.J. (2010). Chemerin: At the crossroads of inflammation and obesity. Trends Endocrinol. Metab..

[7] Bozaoglu K., Bolton K., McMillan J., Zimmet P., Jowett J., Collier G., Walder K., Segal D. (2007). Chemerin Is a Novel Adipokine Associated with Obesity and Metabolic Syndrome. Endocrinology.

[8] Ben Dhaou C., Mandi K., Frye M., Acheampong A., Radi A., De Becker B., Antoine M., Baeyens N., Wittamer V., Parmentier M. (2021). Chemerin regulates normal angiogenesis and hypoxia-driven neovascularization. Angiogenesis.

[9] Zabel B.A., Kwitniewski M., Banas M., Zabieglo K., Murzyn K., Cichy J. (2014). Chemerin regulation and role in host defense. Am. J. Clin. Exp. Immunol..

[10] Spyrou N., Avgerinos K.I., Mantzoros C.S., Dalamaga M. (2018). Classic and Novel Adipocytokines at the Intersection of Obesity and Cancer: Diagnostic and Therapeutic Strategies. Curr. Obes. Rep..

[11] Koliaki C., Liatis S., Dalamaga M., Kokkinos A. (2019). Sarcopenic Obesity: Epidemiologic Evidence, Pathophysiology, and Therapeutic Perspectives. Curr. Obes. Rep..

[12] Sotiropoulos G.P., Dalamaga M., Antonakos G., Marinou I., Vogiatzakis E., Kotopouli M., Karampela I., Christodoulatos G.S., Lekka A., Papavassiliou A.G. (2018). Chemerin as a biomarker at the intersection of inflammation, chemotaxis, coagulation, fibrinolysis and metabolism in resectable non-small cell lung cancer. Lung Cancer.

[13] Zabel B.A., Zuniga L., Ohyama T., Allen S.J., Cichy J., Handel T.M., Butcher E.C. (2006). Chemoattractants, extracellular proteases, and the integrated host defense response. Exp. Hematol..

[14] Wittamer V., Franssen J.D., Vulcano M., Mirjolet J.F., Le Poul E., Migeotte I., Brézillon S., Tyldesley R., Blanpain C., Detheux M. (2003). Specific recruitment of antigen-presenting cells by chemerin, novel processed ligand from human inflammatory fluids. J. Exp. Med..

[15] Shin W.J., Zabel B.A., Pachynski R.K. (2018). Mechanisms and Functions of Chemerin in Cancer: Potential Roles in Therapeutic Intervention. Front. Immunol..

[16] Hart R., Greaves D.R. (2010). Chemerin Contributes to Inflammation by Promoting Macrophage Adhesion to VCAM-1 and Fibronectin through Clustering of VLA-4 and VLA-5. J. Immunol..

[17] Luangsay S., Wittamer V., Bondue B., De Henau O., Rouger L., Brait M., Franssen J.-D., De Nadai P., Huaux F., Parmentier M. (2009). Mouse ChemR23 Is Expressed in Dendritic Cell Subsets and Macrophages, and Mediates an Anti-Inflammatory Activity of Chemerin in a Lung Disease Model. J. Immunol..

[18] Kralisch S., Weise S., Sommer G., Lipfert J., Lossner U., Bluher M., Stumvoll M., Fasshauer M. (2009). Interleukin-1ß induces the novel adipokine chemerin in adipocytes in vitro. Regul. Pept..

[19] Parlee S.D., Ernst M.C., Muruganandan S., Sinal C.J., Goralski K.B. (2010). Serum Chemerin Levels Vary with Time of Day and Are Modified by Obesity and Tumor Necrosis Factor-α. Endocrinol..

[20] Kulig P., Kantyka T., Zabel B.A., Banaś M., Chyra A., Stefańska A., Tu H., Allen S.J., Handel T.M., Kozik A. (2011). Regulation of Chemerin Chemoattractant and Antibacterial Activity by Human Cysteine Cathepsins. J. Immunol..

[21] Banas M., Zabieglo K., Kasetty G., Kapinska-Mrowiecka M., Borowczyk J., Drukala J., Murzyn K., Zabel B.A., Butcher E.C., Schroeder J.M. (2013). Chemerin Is an Antimicrobial Agent in Human Epidermis. PLoS ONE.

[22] Verma D.P., Ansari M.M., Verma N.K., Saroj J., Akhtar S., Pant G., Mitra K., Singh B.N., Ghosh J.K. (2021). Tandem Repeat of a Short Human Chemerin-Derived Peptide and Its Nontoxic d-Lysine-Containing Enantiomer Display Broad-Spectrum Antimicrobial and Antitubercular Activities. J. Med. Chem..

[23] Godlewska U., Bilska B., Zegar A., Brzoza P., Borek A., Murzyn K., Bochenska O., Morytko A., Kuleta P., Kozik A. (2019). The antimicrobial activity of chemerin-derived peptide p4 requires oxidative conditions. J. Biol. Chem..

[24] Godlewska U., Bilska B., Majewski P., Pyza E., Zabel B.A., Cichy J. (2020). Bacteria Modify Their Sensitivity to Chemerin-Derived Peptides by Hindering Peptide Association With the Cell Surface and Peptide Oxidation. Front. Microbiol..

[25] Demoor T., Bracke K.R., Dupont L.L., Plantinga M., Bondue B., Roy M.-O., Lannoy V., Lambrecht B.N., Brusselle G.G., Joos G.F. (2011). The Role of ChemR23 in the Induction and Resolution of Cigarette Smoke-Induced Inflammation. J. Immunol..

[26] Graham K.L., Zabel B.A., Loghavi S., Zuniga L.A., Ho P.P., Sobel R.A., Butcher E.C. (2009). Chemokine-Like Receptor-1 Expression by Central Nervous System-Infiltrating Leukocytes and Involvement in a Model of Autoimmune Demyelinating Disease. J. Immunol..

[27] Cash J., Hart R., Russ A., Dixon J.P., Colledge W.H., Doran J., Hendrick A., Carlton M.B., Greaves D.R. (2008). Synthetic chemerin-derived peptides suppress inflammation through ChemR23. J. Exp. Med..

[28] Weigert J., Obermeier F., Neumeier M., Wanninger J., Filarsky M., Bauer S., Aslanidis C., Rogler G., Ott C., Schäffler A. (2010). Circulating levels of chemerin and adiponectin are higher in ulcerative colitis and chemerin is elevated in Crohnʼs disease. Inflamm. Bowel Dis..

[29] Kukla M., Zwirska-Korczala K., Gabriel A., Waluga M., Warakomska I., Szczygiel B., Berdowska A., Mazur W., Woźniak-Grygiel E., Kryczka W. (2009). Chemerin, vaspin and insulin resistance in chronic hepatitis C. J. Viral Hepat..

[30] Horn P., Metzing U.B., Steidl R., Romeike B., Rauchfuß F., Sponholz C., Thomas-Rüddel D., Ludewig K., Birkenfeld A.L., Settmacher U. (2016). Chemerin in peritoneal sepsis and its associations with glucose metabolism and prognosis: A translational cross-sectional study. Crit. Care.

[31] Ebihara T., Matsumoto H., Matsubara T., Matsuura H., Hirose T., Shimizu K., Ogura H., Kang S., Tanaka T., Shimazu T. (2021). Adipocytokine Profile Reveals Resistin Forming a Prognostic-Related Cytokine Network in the Acute Phase of Sepsis. Shock.

[32] Karampela I., Christodoulatos G.S., Dalamaga M. (2019). The Role of Adipose Tissue and Adipokines in Sepsis: Inflammatory and Metabolic Considerations, and the Obesity Paradox. Curr. Obes. Rep..

[33] Hillenbrand A., Weiss M., Knippschild U., Wolf A.M., Huber-Lang M. (2012). Sepsis-Induced Adipokine Change with regard to Insulin Resistance. Int. J. Inflamm..

[34] Karampela I., Chrysanthopoulou E., Christodoulatos G.S., Dalamaga M. (2020). Is There an Obesity Paradox in Critical Illness? Epidemiologic and Metabolic Considerations. Curr. Obes. Rep..

[35] Hillenbrand A., Xu P., Zhou S., Blatz A., Weiss M., Hafner S., Henne-Bruns D., Knippschild U. (2016). Circulating adipokine levels and prognostic value in septic patients. J. Inflamm..

[36] Karampela I., Christodoulatos G.S., Kandri E., Antonakos G., Vogiatzakis E., Dimopoulos G., Armaganidis A., Dalamaga M. (2019). Circulating eNampt and resistin as a proinflammatory duet predicting independently mortality in critically ill patients with sepsis: A prospective observational study. Cytokine.

[37] Koch A., Weiskirchen R., Krusch A., Bruensing J., Buendgens L., Herbers U., Yagmur E., Koek G.H., Trautwein C., Tacke F. (2018). Visfatin Serum Levels Predict Mortality in Critically Ill Patients. Dis. Markers.

[38] Karampela I., Kandri E., Antonakos G., Vogiatzakis E., Christodoulatos G.S., Nikolaidou A., Dimopoulos G., Armaganidis A., Dalamaga M. (2017). Kinetics of circulating fetuin-A may predict mortality independently from adiponectin, high molecular weight adiponectin and prognostic factors in critically ill patients with sepsis: A prospective study. J. Crit. Care.

[39] Dalamaga M., Karampela I. (2018). Fetuin-A to adiponectin ratio is a promising prognostic biomarker in septic critically ill patients. J. Crit. Care.

[40] Macdonald S.P.J., Bosio E., Neil C., Arendts G., Burrows S., Smart L., Brown S.G.A., Fatovich D. (2017). Resistin and NGAL are associated with inflammatory response, endothelial activation and clinical outcomes in sepsis. Agents Actions.

[41] Karampela I., Dalamaga M. (2021). Serum bilirubin to fetuin-A ratio as a prognostic biomarker in critically ill patients with sepsis. Metab. Open.

[42] Hajri T., Gharib M., Kaul S., Karpeh M.S. (2017). Association between adipokines and critical illness outcomes. J. Trauma Acute Care Surg..

[43] Karampela I., Chrysanthopoulou E., Skyllas G., Christodoulatos G.-S., Kandri E., Antonakos G., Stratigou T., Armaganidis A., Dalamaga M. (2021). Circulating leptin, soluble leptin receptor and free leptin index in critically ill patients with sepsis: A prospective observational study. Minerva Anestesiol..

[44] Bone R.C., Balk R.A., Cerra F.B., Dellinger R.P., Fein A.M., Knaus W.A., Schein R.M., Sibbald W.J. (1992). Definitions for sepsis and organ failure and guidelines for the use of innovative therapies in sepsis. Chest.

[45] Singer M., Deutschman C.S., Seymour C.W., Shankar-Hari M., Annane D., Bauer M., Bellomo R., Bernard G.R., Chiche J.-D., Coopersmith C.M. (2016). The Third International Consensus Definitions for Sepsis and Septic Shock (Sepsis-3). JAMA.

[46] Kassi E., Dalamaga M., Faviou E., Hroussalas G., Kazanis K., Nounopoulos C., Dionyssiou-Asteriou A. (2009). Circulating oxidized LDL levels, current smoking and obesity in postmenopausal women. Atheroscler..

[47] Dalamaga M., Karmaniolas K., Matekovits A., Migdalis I., Papadavid E. (2008). Cutaneous manifestations in relation to immunologic parameters in a cohort of primary myelodysplastic syndrome patients. J. Eur. Acad. Dermatol. Venereol..

[48] Papadavid E., Gazi S., Dalamaga M., Stavrianeas N., Ntelis V. (2008). Palmoplantar and scalp psoriasis occurring during anti–tumour necrosis factor-α therapy: A case series of four patients and guidelines for management. J. Eur. Acad. Dermatol. Venereol..

[49] Dalamaga M., Christodoulatos G.S. (2015). Adiponectin as a biomarker linking obesity and adiposopathy to hematologic malignancies. Horm. Mol. Biol. Clin. Investig..

[50] Dalamaga M., Karmaniolas K., Arsenis G., Pantelaki M., Daskalopoulou K., Papadavid E., Migdalis I. (2008). Cedecea lapagei bacteremia following cement-related chemical burn injury. Burns.

[51] Dalamaga M., Nikolaidou A., Karmaniolas K., Hsi A., Chamberland J., Dionyssiou-Asteriou A., Mantzoros C.S. (2007). Circulating Adiponectin and Leptin in Relation to Myelodysplastic Syndrome: A Case-Control Study. Oncol..

[52] Knaus W.A., Draper E.A., Wagner D.P., Zimmerman J.E. (1985). APACHE II: A severity of disease classification system. Crit. Care Med..

[53] Conde J., Gomez R., Bianco G., Scotece M., Lear P., Dieguez C., Gomez-Reino J., Lago F., Gualillo O. (2011). Expanding the adipokine network in cartilage: Identification and regulation of novel factors in human and murine chondrocytes. Ann. Rheum. Dis..

[54] Monnier J., Lewén S., O’Hara E., Huang K., Tu H., Butcher E.C., Zabel B.A. (2012). Expression, Regulation, and Function of Atypical Chemerin Receptor CCRL2 on Endothelial Cells. J. Immunol..

[55] Du X.-Y., Zabel B.A., Myles T., Allen S.J., Handel T.M., Lee P.P., Butcher E.C., Leung L.L. (2009). Regulation of Chemerin Bioactivity by Plasma Carboxypeptidase N, Carboxypeptidase B (Activated Thrombin-activable Fibrinolysis Inhibitor), and Platelets. J. Biol. Chem..

[56] Tan B.K., Chen J., Farhatullah S., Adya R., Kaur J., Heutling D., Lewandowski K.C., O’Hare J.P., Lehnert H., Randeva H.S. (2009). Insulin and Metformin Regulate Circulating and Adipose Tissue Chemerin. Diabetes.

